# Postpartum Endomyometritis

**DOI:** 10.1155/S1064744995000640

**Published:** 1995

**Authors:** Katherine L. Williams, Joseph G. Pastorek II

**Affiliations:** Department of Obstetrics and Gynecology Louisiana State University Medical Center 1542 Tulane Avenue Near Orleans LA 70112-2822 USA

## Abstract

Endomyometritis following parturition is a major cause of maternal morbidity. It is most common following cesarean delivery, especially in certain high-risk patient populations. The infection is usually caused by bacteria in the cervicovaginal tract that are inoculated into the uterus during labor and delivery. Both anaerobes and aerobes are thought to be involved in the disease process. A prompt diagnosis based on clinical suspicion, a thorough physical examination, and adjunctive laboratory measures is necessary to insure effective therapy and prompt resolution of the infection. The treatment consists of supportive care and broad-spectrum antibiotic coverage either with single extended-spectrum drugs or with combinations of antimicrobials. In cases appropriately treated, recovery without sequelae is the rule.


Infectious Diseases in Obstetrics and Gynecology 3:210–216 (1995)
© 1996 Wiley-Liss, Inc.

### Postpartum Endomyometritis

**Katherine L. Williams and Joseph G. Pastorek II**

*Department of Obstetrics and Gynecology, Louisiana State University Medical Center, New Orleans, LA*

ABSTRACT

**Endomyometritis following parturition is a major cause of maternal morbidity. It is most common following cesarean delivery, especially in certain high-risk patient populations. The infection is usually caused by bacteria in the cervicovaginal tract that are inoculated into the uterus during labor and delivery. Both anaerobes and aerobes are thought to be involved in the disease process. A prompt diagnosis based on clinical suspicion, a thorough physical examination, and adjunctive laboratory measures is necessary to insure effective therapy and prompt resolution of the infection. The treatment consists of supportive care and broad-spectrum antibiotic coverage either with single extended-spectrum drugs or with combinations of antimicrobials. In cases appropriately treated, recovery without sequelae is the rule.** © 1996 Wiley-Liss, Inc.

KEY WORDS

Puerperal infection, female pelvic infection, antibiotic therapy, endometritis, endomyoparametritis

Postpartum endometritis or, more properly, endomyometritis is one of the most common female pelvic infections and often a serious and occasionally fatal complication of delivery. This puerperal infection is named depending upon the extent of the disease process. *Endometritis* is defined as an inflammation of the endometrium that is present universally after parturition; *endomyometritis* implies clinically significant infectious involvement of the endometrium and myometrium; and *endomyoparametritis* represents an extension of the infection to the parametrium. Of course, an extension of the infection into the peritoneal cavity generally results in peritonitis, perhaps intra-abdominal abscess, and generalized sepsis.

Endometritis is 7–30 times more likely to occur after an abdominal delivery, hence, the commonly encountered terms *postcesarean endometritis* and *postcesarean endomyometritis*. There is a 5–6% incidence of the infection after elective cesarean deliveries in a low-risk "private" population and a 22–85% incidence after nonelective procedures, especially in high-risk indigent patients in teaching hospitals. Prophylactic antibiotic coverage decreases the latter incidence by 50–60%.[1] Adolescents are reported to have a 2:1 greater incidence of postpartum endomyometritis compared with the overall prevalence among adults, 23% vs. 11%, respectively, perhaps attributable to the higher prevalence rates of various sexually transmitted diseases among the younger population.[2]

### RISK FACTORS

Several factors put a parturient at an increased risk of endomyometritis (Table 1). The duration of labor, number of cervical examinations, and prolonged rupture of the membranes are interrelated, subject to obstetric complications such as cephalopelvic disproportion. Rupture of the membranes for >6 h has been associated with a 95% incidence of infection.[3]

The fact that postpartum endomyometritis is more common in human immunodeficiency virus (HIV)-infected women (an incidence of approximately 10.3% vs. 4.2% in seronegative controls in one preliminary study) demonstrates that compro-

Address correspondence/reprint requests to Dr. Joseph G. Pastorek II, Department of Obstetrics and Gynecology, Louisiana State University Medical Center, 1542 Tulane Avenue, New Orleans, LA 70112-2822.

Received September 6, 1995
Accepted December 18, 1995

Review Article

**TABLE 1. Risk factors for the development of puerperal endomyometritis**

| |
|---|
| Operative delivery, especially cesarean |
| Prolonged duration of labor |
| Prolonged rupture of membranes |
| Number of cervical examinations |
| Immune status |
| Blood loss or anemia |
| Obesity |
| Maternal age, especially young |
| Amnionitis or amniotic-fluid colonization |
| Antepartum infection or colonization by group B streptococcus or gonorrhea |
| Intrapartum bacteriuria |
| Low socioeconomic status |
| Preterm gestational age |

mised immunity is a risk. Moreover, the $CD_4$ cell population percentage is inversely correlated with the incidence of postpartum endomyometritis.[4] Nevertheless, further study of HIV-infected gravidas is indicated because of the methodologic flaws in this study which need to be corrected in larger trials.

In one study in which obesity was defined as a body weight >136 kg, postpartum endomyometritis occurred in 45% of the obese patients, while no cases were reported in the control group. However, the incidence of cephalopelvic disproportion was more common, thereby increasing the likelihood of an abdominal delivery and other risk factors for endomyometritis.[5]

An unrecognized infection of the urogenital tract is a significant independent prediction of postpartum endometritis. Bacteria such as *Ureaplasma urealyticum* are capable of migrating across the intact amnion and multiplying if the bacteriostatic activity of the amniotic fluid is insufficient. This colonization of the chorioamnion allows for the subsequent colonization of the endometrium.[6] Furthermore, infection has been implicated in the pathogenesis of preterm delivery, consequently establishing a relationship between preterm gestational age and postpartum endomyometritis.[7] Finally, intrapartum bacteriuria has been associated with endomyometritis in women who delivery vaginally.[8] It appears reasonable to assume that, with the manipulation entailed in vaginal delivery, even a subclinical bacterial colonization of the bladder could result in dissemination. Thus, the early identification of such infections may help prevent the development of endometritis.

Risk factors inherent to cesarean delivery include breaks in the sterile techniques,[9] a resident staff member as the primary surgeon, an extension of the uterine incision, and positive endometrial cultures at the time of the operation.[10] Additionally, elective repeat cesarean deliveries have less associated febrile morbidity than other cesarean deliveries because of the lack of bacterial contamination of the lower uterine segment (the incisional area) by the forces of labor and the effacement of the cervix.[10,11]

### PATHOPHYSIOLOGY

In order for bacteria to colonize and invade susceptible tissue, any 1 of 3 factors must usually be present: 1) weakened host defense mechanisms, e.g., trauma and disruption of the normal mechanical barriers to bacterial invasion; 2) an impaired host immunity secondary to an immunocompromising illness, medications, or other less evident factors such as inadequate nutrition; or 3) the particular virulence or invasiveness of the bacteria in question. Endomyometritis is usually thought to be an ascending infection, progressing from the lower uterine segment contaminated by the cervicovaginal flora to the uterine fundus and finally the peritoneal cavity.[12] The site of infection in the untraumatized uterus is generally assumed to be the denuded implantation site, which is usually high in the fundus far from the contaminated lower uterine segment.[12] Postpartum endomyometritis would, therefore, be considerably less common after a vaginal delivery than after a cesarean delivery, in which the most vulnerable area is the suture line in the contaminated lower uterine segment.[13]

There are 2 different clinical presentations of puerperal endomyometritis, with much overlap between them. Late-onset endomyometritis, which may be more common after vaginal delivery than after cesarean delivery, is considered to be an infection with an onset of 2 days to 6 weeks after delivery.[14] With late-onset disease, the patient is often afebrile with only mild symptoms.[13] This presentation of the disease is thought to be associated with certain isolates such as *Chlamydia trachomatis*, the genital mycoplasmas, and, to a lesser extent, anaerobic bacteria.[13,15]

Early-onset postpartum endomyometritis is more often associated with a cesarean delivery. It develops within 48 h of delivery due to the contami-

TABLE 2. Other possible causes of puerperal fever

| |
|---|
| Wound infection |
| Pyelonephritis |
| Cystitis |
| Pneumonia |
| Atelectasis |
| IV-site phlebitis |
| Breast engorgement |
| Drug fever |
| Miscellaneous medical and surgical diseases |

nation of the endometrial cavity with vaginal organisms during labor and delivery.[15] The damaged, sutured tissue around the incision site is more susceptible to bacterial invasion, which is usually polymicrobial with a predominance of anaerobic pathogens.[3] Furthermore, the collection of serum and blood accumulating around the suture site is a good medium for microbial growth.[12] Incidentally, no increased incidence of endomyometritis has been noted with the classical cesarean incision vs. the low transverse incision.[16]

### DIAGNOSES

The diagnosis of postpartum endomyometritis is generally made on the basis of fever, uterine tenderness, and the exclusion of other causes or sites of infection. Fever and tachycardia may be the 2 earliest signs of infection. Fever is defined as an oral temperature of ≤38°C on 2 consecutive readings 6 h apart, 24 h after delivery, or a temperature ≥38.3°C at any time after delivery. Uterine tenderness with or without peritonitis and foul-smelling lochia are also important diagnostic parameters. A left shift in the WBC differential, although nonspecific, should raise suspicion. If one or more of these criteria are present, further evaluation, in other words, the "fever workup," is required.[9] Other ancillary findings may include a pelvic mass or tenderness or induration of the adnexal areas.[12]

A focused physical examination should be performed to exclude other causes of puerperal infection (Table 2). A wound infection would be suspected in a case of erythema, induration, and discharge associated with the sutured incision, be it abdominal or episiotomy. Costovertebral angle tenderness and dysuria are suggestive of pyelonephritis, while suprapubic tenderness, dysuria, and a low-grade temperature with a normal pulse are characteristic of cystitis. Abnormal lung auscultation with symptoms of respiratory distress may indicate a pneumonia or atelectasis.[12]

When examining the lower abdomen and pelvis, the physician should palpate areas away from the uterine and abdominal incisions to avoid false positives representing "normal" surgical tenderness. The uterus should be approached from the cephalad direction, above the umbilicus, to encounter the fundus well above the usual cesarean incision. The physician should also palpate the parametria for tenderness in a gentle but deep manner, keeping in mind that a patient who has had a bilateral tubal ligation will have tenderness in this area.[12]

The speculum examination should include an inspection for foul-smelling or purulent lochia as well as retained lochia secondary to an improperly draining cervical os (the so-called "lochial block").[12] In addition, cultures should be obtained. A double-lumen catheter technique is considered the most clinically useful and effective in reducing any contamination of the specimen with organisms of the lower genital tract.[17] However, an extended sterile swab from as high in the uterine fundus as possible to avoid cervical contamination, which is considered more cost effective, has proven to produce a good representative sample of the infected endometrium.[18] It should be remembered that a culture of the amniotic fluid or placental surfaces at the time of cesarean delivery would allow a much earlier identification of the potential pathogenic microbes.[19]

There is some debate as to the utility of endometrial cultures in the course of caring for the patient with endomyometritis. One philosophy holds that the cultures are basically useless except to a research setting because the patient will probably be discharged and cured by the time the final results are available. On the other hand, even in a nonresearch environment, a serial identification of the organisms responsible for the infection gives the physician and the institution a data base upon which to formulate future antibiotic choices. If no cultures are taken, any shifts in the flora or changes in the antibiotic sensitivities within a given hospital or center will go unnoticed. For the individual patient failing her initial therapy, preliminary culture results may often be of help in the selection of replacement antibiotics. For example, in a patient initially treated with a cephalosporin or cephamycin who is not recovering as expected, the finding of a

TABLE 3. Common bacterial isolates in cases of puerperal endomyometritis

| Gram negative | Gram positive | Anaerobes |
|---|---|---|
| *Escherichia coli* | Enterococci | Bacteroides[a] |
| *Klebsiella* sp. | Other streptococci | *Clostridium* sp. |
| *Enterobacter* sp. | Staphylococci | *Veilonella* sp. |
| *Proteus* sp. | Anaerobic peptococci | *Fusobacterium* sp. |

[a]Including genera now classified as *Bacteroides* and *Prevotella*.

heavy growth of a gram-positive coccus (possibly enterococcus) would dictate a different antibiotic switch than would the finding of a gram-negative rod (*Pseudomonas* perhaps) in the endometrial culture plates.

The next step in the scheme of patient evaluation is a bimanual examination to assess any elevated pelvic temperature as well as any abnormal masses or fullness. An outline of the uterus, paremetrial areas, and especially the anterior uterine surface in the area of the uterine incision in an effort to delineate any abnormal fluid collections completes a thorough pelvic examination.[12]

Finally, in addition to a complete blood count and endometrial cultures, the laboratory evaluation should include a urinalysis and urine culture of a catheterized specimen, electrolytes, and blood cultures. Blood cultures may be expected to be positive in only 5–20% of the gravidas with endomyometritis; however, the identification of virulent organisms in the blood such as *Staphylococcus aureus* may have implications in the patient's therapy.

### BACTERIOLOGY

Endomyometritis is considered to be a mixed infection usually involving multiple isolates, both aerobic and anaerobic, although sometimes it involves a predominant organism.[12,20] Since postpartum endomyometritis is an upper-genital-tract infection involving bacteria from the cervicovaginal flora, the pathogens frequently encountered are common to the latter, such as *Gardnerella vaginalis*, *Prevotella bivia* (formerly *Bacteroides bivius*), and *Escherichia coli*.[21] However, antibiotic coverage should be provided for *Streptococcus agalactiae* (the group B streptococcus)[21] and, in light of recent attention, *Enterococcus fecaelis*.[22] Further, in postoperative patients receiving β-lactam prophylaxis, there is an increased association of gram-positive isolates and nonfragilis strains of *Bacteroides*.[21] Table 3 provides a partial list of organisms that have been associated with postpartum endomyometritis, particularly early-onset or postcesarean disease.

TABLE 4. Antibiotic regimens for the treatment of puerperal endomyometritis

| | |
|---|---|
| Combination therapy | |
| | Clindamycin plus an aminoglycoside[a] |
| | Metronidazole plus an aminoglycoside |
| | Ampicillin plus metronidazole plus an aminoglycoside |
| | Clindamycin plus aztreonam |
| Single-agent therapy | |
| | Cefoxitin |
| | Cefotetan |
| | Cefmetazole |
| | Cefotaxime |
| | Ceftizoxime |
| | Mezlocillin[b] |
| | Ticarcillin[b] |
| | Piperacillin[b] |
| | Ticarcillin/clavulanate |
| | Piperacillin/tazobactam |
| | Ampicillin/sulbactam |

[a]Gentamicin or tobramycin most commonly used, based upon cost.
[b]Not recommended in areas where bacterial β-lactamase is common.

### TREATMENT

The recommended antibiotic therapy for puerperal endomyometritis usually includes a 2nd- or 3rd-generation cephalosporin, an expanded-spectrum penicillin, or a combination of antibiotics, offering broad coverage of the mixed bacterial flora participating in the disease (Table 4). Intravenous (IV) antibiotics are usually continued until the patient has been afebrile for 24–48 h. Clindamycin plus an aminoglycoside (gentamicin or tobramycin), often termed the "gold standard" of endometritis therapy, has been listed most extensively, with a cure rate of over 90%.[23] With the greater volume of distribution and rapid elimination in the postpartum patient, the therapeutic levels of aminoglycosides are seldom achieved. Practitioners often claim that achieving a clinical response is more important than achieving the standard therapeutic levels. However, care must be taken, as aminoglycosides have a propensity for renal toxicity and ototoxicity.[24]

Broad-spectrum cephalosporins such as cefoxitin, cefotetan, and moxalactam as well as semisynthetic penicillins such as ticarcillin, piperacillin, and mezlocillin with the addition of a β-lactam inhibitor have also proved successful in the treatment of endomyometritis. In general, the reports of single-agent therapy for puerperal endomyometritis have demonstrated equal efficacy compared with the use of combinations of drugs.[23,25]

If a patient continues to spike a temperature after 72 h of any of the aforementioned treatments, she should be changed to triple antibiotic coverage. Ampicillin, gentamicin, and clindamycin are common choices to increase the spectrum of coverage and to provide defense against resistant organisms. If a patient remains febrile after an additional 72 h of coverage, other complications such as a deep venous thrombosis and pelvic abscess must be excluded.

After a patient has been afebrile for 24–48 h, IV antibiotics can be discontinued. The need for continuing therapy with an oral antibiotic has been disputed, but no increase in morbidity has been found in patients discharged without oral medications.[26,27]

Some patients may require extended treatment and oral equivalents for such problems as bacteremia, limited IV access, pelvic abscess, septic pelvic thrombophlebitis, and pyelonephritis. Some oral agents commonly used in these circumstances include ampicillin, which is suitable for enterococcal infection but poor against many of the Enterobacteriaceae, anaerobes, and staphylococcal species. Metronidazole has excellent anaerobic coverage, but not against most gram-positive organisms and gram-negative aerobic bacteria. Cephalosporins, however, have good gram-negative activity and reasonable gram-positive activity. Quinolones are comparable to other oral agents; they have excellent activity against gram-negative aerobic and facultative organisms, as well as good gram-positive activity. However, the quinolones as a group have only moderate activity against anaerobes.[21] Finally, erythromycin has proved to be an effective oral agent in late-onset postpartum endometritis.[14]

Other therapeutic measures should include hydration and abdominal decompression. A patient should not have any oral intake if she has peritonitis or an abdominal ileus. In such a patient, nasogastric suctioning may be required. The electrolyte balance is also important. Hypokalemia aggravates an already compromised intestinal motility. Pulmonary toilet is often required secondary to the poor inspiratory effort, especially associated with abdominal distention or peritonitis, which can lead to atelectasis and decreased arterial oxygen saturation. Blood replacement may be necessary if the hematocrit is <30 to help enhance oxygenation, especially if the patient is symptomatic. Wound care consists of heat application for abdominal-wound cellulitis; opening and drainage of an abscess or hematoma; drainage, debridement, and repair of compromised fascia; and treatment of open wounds with wet-to-dry dressing changes with 0.25% acetic acid or normal saline 3 times a day. Finally, the uterus should be evacuated if retained products are suspected.[12]

### PROPHYLAXIS

Antibiotic prophylaxis to prevent endomyometritis is commonly used in high-risk patients undergoing cesarean delivery, e.g., patients with prolonged labor or prolonged rupture of the membranes. Prophylactic agents can be instituted antepartally or perioperatively. The routine use of antibiotics has proved to be beneficial in high-risk obstetric patients with postpartal endometritis. In 460 women in the 3rd trimester in labor given a single dose of ceftriaxone, 250 mg intramuscularly (IM), the incidence of postpartum endometritis was 3.8% vs. 10.4% in the placebo group.[28]

There is universal agreement on the use of antibiotic prophylaxis for high-risk patients undergoing cesarean birth. However, its use in low-risk populations is debated. Yet, recent research has shown that a prophylactic dose of 1 g of cefazolin is both beneficial and cost effective. Febrile morbidity decreased from a 17.9% prevalence in the control group to approximately 9%.[29] Objections have been made regarding the potential for resistant organisms and side effects; however, bacterial resistance is less likely to occur after a single dose than after multiple doses.[9,30] Furthermore, prophylaxis failure has been attributed to incipient infection at the time of delivery that may limit the effectiveness of the prophylaxis; in other words, the patient needs early *therapy*, not prophylaxis. Thus, the early identification of patients with underlying infections will allow a timely institution of therapeutic antibiotics.[31]

The type of agent used prophylactically at cesarean delivery has been widely discussed. Generally speaking, no benefit is derived from using more than a single dose of an antibiotic in most cases. The choice of drug (1st-generation vs. 2nd-generation vs. 3rd-generation cephalosporin) seems to be unimportant. Cost is usually the deciding factor within a given institution as to which medication is preferred for surgical prophylaxis.

### SEQUELAE

Investigators have placed much emphasis on the treatment of puerperal endomyometritis in hopes of avoiding the serious complications that can occur. Bacteremia has been documented in 8–20% of the cases of postcesarean endometritis and in 5% of patients after vaginal delivery. The antibiotic failure rates and complications are also higher in the cesarean-delivery populations.[1] Bacteremic patients often have temperatures that can become alarmingly high or persist for a prolonged period. Anaerobes are often the culprits, with *Bacteroides fragilis* usually causing the highest fever index.[32] However, a specific syndrome of high spiking temperature within hours of delivery has been attributed to the group B streptococcus.[33] Generally speaking, though, if a bacteremic patient responds to antibiotics in a timely fashion, she is treated in the same manner as a nonbacteremic patient with otherwise similar disease. The only exceptions may be an individual with cardiac or other indwelling prostheses or a patient with bacteremia due to *S. aureus*. In these latter cases, a more prolonged course of oral antibiotics may need to be prescribed after the patient's discharge.

Pelvic abscess and septic pelvic thrombophlebitis have a similar prevalence: 4.9% after cesarean-associated endometritis and 1.9% after endometritis following vaginal delivery. Regarding fertility, neither cesarean-associated endometritis nor pelvic cellulitis affects the rate of subsequent pregnancy. However, a pelvic abscess decreases the future fertility rate by approximately 50%.[11] Death from sepsis is 81 times more likely to occur after cesarean delivery.[1]

In summary, an identification of the risk factors should be the physician's first step in the management of postpartum endometritis. A postcesarean or obese patient should prompt close observation for any signs of infection. A measure of suspicion along with a thorough physical examination that reveals extreme tenderness and symptoms of infection should prompt the clinician to obtain cultures and initiate treatment. Furthermore, early treatment is both efficacious and cost effective in preventing the serious complications that can ensue.

### REFERENCES

1. Yonekura ML: Treatment of postcesarean endomyometritis. Clin Obstet Gynecol 31:488–500, 1988.
2. Berenson AB, Hammill HA, Martens MG, Faro S: Bacteriologic findings of post-cesarean endometritis in adolescents. Obstet Gynecol 75:627–629, 1990.
3. Gilstrap LC, Cunningham FG: The bacterial pathogenesis of infection following cesarean section. Obstet Gynecol 53:545–549, 1979.
4. Temmerman M, Chomba E, Ndinya-Achola J, et al.: Maternal human immunodeficiency virus-1 infection and pregnancy outcome. Obstet Gynecol 83:495–501, 1994.
5. Isaacs, JD, Magann EF, Martin RN, et al.: Obstetric challenges of massive obesity complicating pregnancy. J Perinatol 14:10–14, 1994.
6. Andrews WW, Shah SR, Goldenberg RL, et al.: Association of post-cesarean delivery endometritis with colonization of the chorioamnion by *Ureaplasma urealyticum*. Obstet Gynecol 85:509–514, 1995.
7. Seo K, McGregor JA, French JI: Preterm birth is associated with increased risk of maternal and neonatal infection. Obstet Gynecol 79:75–80, 1992.
8. Monif GRG: Intrapartum bacteriuria and postpartum endometritis. Obstet Gynecol 78:245–248, 1991.
9. Martens MG, Faro S, Phillips LE, Poindexter AN: Postpartum endometritis in high risk c-section patients. Infect Surg 6:96–99, 1987.
10. Repke JT, Spence MR, Calhoun S: Risk factors in the development of cesarean section infection. Surg Gynecol Obstet 158:112–116, 1984.
11. Hurry DJ, Larsen B, Charles D: Effects of postcesarean section febrile morbidity on subsequent fertility. Obstet Gynecol 64:256–260, 1984.
12. Pastorek JG, Miller JM: Postcesarean section infection. Infect Surg 6:532–544, 1987.
13. Hoyme UB, Kiviat N, Eschenbach DA: Microbiology and treatment of late postpartum endometritis. Obstet Gynecol 68:226–232, 1986.
14. Rosen K, Eschenbach DA, Tomplins LS, et al.: Polymicrobial early postpartum endometritis with facultative and anaerobic bacteria, genital mycoplasmas and *Chlamydia trachomatis:* Treatment with piperacillin or cefoxitin. J Infect Dis 153:1028–1037, 1986.
15. Watts DH, Eschenbach DA, Kenny GE: Early postpartum endometritis: The role of bacteria, genital mycoplasmas and *Chlamydia trachomatis*. Obstet Gynecol 73:52–60, 1989.
16. Blanco JD, Gibbs RS: Infections following classical cesarean section. Obstet Gynecol 55:167–169, 1980.
17. Duff P, Gibbs R, Blanco J, St. Clair P: Endometrial culture techniques in puerperal patients. Obstet Gynecol 61:217, 1983.
18. Martens MG, Faro S, Hammill H, et al.: Comparison of two endometrial sampling devices, cotton-tipped swab and double-lumen catheter with a brush. J Reprod Med 34:875–879, 1989.
19. Cooperman NR, Kasim M, Rajashekaraiah KR: Clinical significance of amniotic fluid, amniotic membranes and endometrial biopsy culture at the time of cesarean section. Am J Obstet Gynecol 37:536, 1980.
20. Martens MG, Faro S, Hammill HA, et al.: Transcervical uterine cultures with a new endometrial suction curette: A comparison of three sampling methods in postpartum endometritis. Obstet Gynecol 74:273–276, 1989.
21. Martens MG, Faro S, Maccato M, et al.: Susceptibility of female pelvic pathogens to oral antibiotic agents in patients who develop postpartum endometritis. Am J Obstet Gynecol 164:1383–1386, 1991.
22. Faro S, Pastorek JG: The enterococcus: Myth or reality in ob/gyn infections? Infect Surg 2:787–794, 1983.
23. McGregor JA, Crombleholme NR, Newton E, et al.: Randomized comparison of ampicillin-sulbactam to cefoxitin and doxycycline or clindamycin and gentamicin in the treatment of pelvic inflammatory disease or endometritis. Obstet Gynecol 83:998–1004, 1994.
24. Letterie GS: Serum gentamicin concentrations in postpartum endometritis. Mil Med 157:526–529, 1992.
25. Pastorek JG, Sanders CV: Antibiotic therapy for postcesarean endomyometritis. Rev Infect Dis 13(Suppl 9): S752–757, 1991.
26. Soper DE, Kemmer CT, Conover WB: Abbreviated antibiotic therapy for the treatment of postpartum endometritis. Obstet Gynecol 69:127–130, 1987.
27. Hager WD, Pascuzzi M, Vernon M: Efficacy of oral antibiotics following parenteral antibiotics for serious infection in obstetrics and gynecology. Obstet Gynecol 73:326–329, 1989.
28. Temmerman M, Njagi E, Nagelkerke N, et al.: Mass antimicrobial treatment in pregnancy: A randomized placebo-controlled trial in a population with high rates of sexually transmitted diseases. J Reprod Med 40:176–180, 1995.
29. Jakobi P, Neissman A, Sigler E, et al.: Postcesarean section febrile morbidity antibiotic prophylaxis in low-risk patients. J Reprod Med 39:707–710, 1994.
30. Kennedy R, Duncan D: Antibiotic prophylaxis in obstetrics and gynecology. In Sweet RL, Gibbs RS (eds): Infectious Diseases of the Female Genital Tract. 2nd ed. Baltimore: Williams & Wilkins, pp 460–473, 1990.
31. Gonik B, Shannon R, Shawar R, et al.: Why patients fail antibiotic prophylaxis at cesarean delivery: Histologic evidence for incipient infection. Obstet Gynecol 79:179–183, 1992.
32. Dizerega GS, Yonekura ML, Leegan K, et al.: Bacteremia in post-cesarean endomyometritis: Differential response to therapy. Obstet Gynecol 55:587–590, 1980.
33. Faro S: Group B streptococcus and puerperal sepsis. Am J Obstet Gynecol 138:1219–1220, 1980.

## References

[PDF_00551] Andrews W. W., Shah S. R., Goldenberg R. L., Cliver S. P., Hauth J. C., Cassell G. H. (1995). Association of post-cesarean delivery endometritis with colonization of the chorioamnion by Ureaplasma urealyticum.. Obstet Gynecol.

[PDF_00538] Berenson A. B., Hammill H. A., Martens M. G., Faro S. (1990). Bacteriologic findings of post-cesarean endometritis in adolescents.. Obstet Gynecol.

[PDF_00583] Blanco J. D., Gibbs R. S. (1980). Infections following classical cesarean section.. Obstet Gynecol.

[PDF_00592] Cooperman N. R., Kasim M., Rajashekaraiah K. R. (1980). Clinical significance of amniotic fluid, amniotic membranes, and endometrial biopsy cultures at the time of cesarean section.. Am J Obstet Gynecol.

[PDF_00641] DiZerega G. S., Yonekura M. L., Keegan K., Roy S., Nakamura R., Ledger W. (1980). Bacteremia in post-Cesarean section endomyometritis: differential response to therapy.. Obstet Gynecol.

[PDF_00585] Duff P., Gibbs R. S., Blanco J. D., St Clair P. J. (1983). Endometrial culture techniques in puerperal patients.. Obstet Gynecol.

[PDF_00644] Faro S. (1980). Group B streptococcus and puerperal sepsis.. Am J Obstet Gynecol.

[PDF_00541] Gilstrap L. C., Cunningham F. G. (1979). The bacterial pathogenesis of infection following cesarean section.. Obstet Gynecol.

[PDF_00621] Hager W. D., Pascuzzi M., Vernon M. (1989). Efficacy of oral antibiotics following parenteral antibiotics for serious infections in obstetrics and gynecology.. Obstet Gynecol.

[PDF_00571] Hoyme U. B., Kiviat N., Eschenbach D. A. (1986). Microbiology and treatment of late postpartum endometritis.. Obstet Gynecol.

[PDF_00566] Hurry D. J., Larsen B., Charles D. (1984). Effects of postcesarean section febrile morbidity on subsequent fertility.. Obstet Gynecol.

[PDF_00548] Isaacs J. D., Magann E. F., Martin R. W., Chauhan S. P., Morrison J. C. (1994). Obstetric challenges of massive obesity complicating pregnancy.. J Perinatol.

[PDF_00630] Jakobi P., Weissman A., Sigler E., Margolis K., Zimmer E. Z. (1994). Post-cesarean section febrile morbidity. Antibiotic prophylaxis in low-risk patients.. J Reprod Med.

[PDF_00613] Letterie G. S. (1992). Serum gentamicin concentrations in postpartum endomyometritis.. Mil Med.

[PDF_00596] Martens M. G., Faro S., Hammill H. A., Riddle G. D., Smith D. (1989). Transcervical uterine cultures with a new endometrial suction curette: a comparison of three sampling methods in postpartum endometritis.. Obstet Gynecol.

[PDF_00588] Martens M. G., Faro S., Hammill H., Phillips L. E., Riddle G. D. (1989). Comparison of two endometrial sampling devices. Cotton-tipped swab and double-lumen catheter with a brush.. J Reprod Med.

[PDF_00602] Martens M. G., Faro S., Maccato M., Riddle G., Hammill H. A. (1991). Susceptibility of female pelvic pathogens to oral antibiotic agents in patients who develop postpartum endometritis.. Am J Obstet Gynecol.

[PDF_00608] McGregor J. A., Crombleholme W. R., Newton E., Sweet R. L., Tuomala R., Gibbs R. S. (1994). Randomized comparison of ampicillin-sulbactam to cefoxitin and doxycycline or clindamycin and gentamicin in the treatment of pelvic inflammatory disease or endometritis.. Obstet Gynecol.

[PDF_00558] Monif G. R. (1991). Intrapartum bacteriuria and postpartum endometritis.. Obstet Gynecol.

[PDF_00563] Repke J. T., Spence M. R., Calhoun S. (1984). Risk factors in the development of cesarean section infection.. Surg Gynecol Obstet.

[PDF_00574] Rosene K., Eschenbach D. A., Tompkins L. S., Kenny G. E., Watkins H. (1986). Polymicrobial early postpartum endometritis with facultative and anaerobic bacteria, genital mycoplasmas, and Chlamydia trachomatis: treatment with piperacillin or cefoxitin.. J Infect Dis.

[PDF_00555] Seo K., McGregor J. A., French J. I. (1992). Preterm birth is associated with increased risk of maternal and neonatal infection.. Obstet Gynecol.

[PDF_00618] Soper D. E., Kemmer C. T., Conover W. B. (1987). Abbreviated antibiotic therapy for the treatment of postpartum endometritis.. Obstet Gynecol.

[PDF_00544] Temmerman M., Chomba E. N., Ndinya-Achola J., Plummer F. A., Coppens M., Piot P. (1994). Maternal human immunodeficiency virus-1 infection and pregnancy outcome.. Obstet Gynecol.

[PDF_00625] Temmerman M., Njagi E., Nagelkerke N., Ndinya-Achola J., Plummer F. A., Meheus A. (1995). Mass antimicrobial treatment in pregnancy. A randomized, placebo-controlled trial in a population with high rates of sexually transmitted diseases.. J Reprod Med.

[PDF_00579] Watts D. H., Eschenbach D. A., Kenny G. E. (1989). Early postpartum endometritis: the role of bacteria, genital mycoplasmas, and Chlamydia trachomatis.. Obstet Gynecol.

[PDF_00536] Yonekura M. L. (1988). Treatment of postcesarean endomyometritis.. Clin Obstet Gynecol.

